# Exosomal miRNA expression profiling in patients with imatinib resistant Chronic myeloid leukemia: A pilot study

**DOI:** 10.1371/journal.pone.0331479

**Published:** 2025-08-29

**Authors:** Raphatphorn Navakanitworakul, Pirun Saelue, Tipparat Penglong, Piyatida Molika, Natakorn Nokchan, Natta Tansila, Hansuk Buncherd, Supinya Thanapongpichat, Kanitta Srinoun

**Affiliations:** 1 Department of Biomedical Sciences and Biomedical Engineering, Faculty of Medicine, Prince of Songkla University, Songkhla, Thailand; 2 Hematology Unit, Division of Internal Medicine, Faculty of Medicine, Prince of Songkla University, Hatyai, Songkhla, Thailand; 3 Department of Pathology, Faculty of Medicine, Prince of Songkla University, Hat Yai, Songkhla, Thailand; 4 Translational Medicine Research Center, Faculty of Medicine, Prince of Songkla University, Songkhla, Thailand; 5 Faculty of Medical Technology, Prince of Songkla University, Hat Yai, Songkhla, Thailand; Tarbiat Modares University, IRAN, ISLAMIC REPUBLIC OF

## Abstract

Chronic myeloid leukemia (CML) is a hematologic malignancy originating from hematopoietic stem cells and driven by the BCR-ABL fusion oncogene. Imatinib (IM), a tyrosine kinase inhibitor, is commonly used as a frontline therapy for CML. However, some patients exhibit primary resistance or show persistent molecular evidence of disease despite treatment. Emerging studies indicate that exosome-derived microRNAs (miRNAs) play a role in mediating drug resistance and may serve as promising biomarkers for cancer diagnosis and predicting therapeutic response. This study aimed to investigate the plasma exosomal miRNA expression profiles in CML patients to identify potential biomarkers associated with IM resistance. Exosomes were isolated from plasma samples of both IM-sensitive and IM-resistant CML patients. The exosomal miRNA content was analyzed using RNA sequencing, followed by differential expression analysis, which revealed 13 upregulated and 21 downregulated miRNAs in IM-resistant patients. Subsequent bioinformatics analysis indicated significant enrichment in pathways related to autophagy and PI3K-Akt signaling. Notably, miR-451a and miR-16–2-3p were among the most significantly upregulated miRNAs in exosomes from IM-resistant individuals. Interestingly, miR-16–2-3p expression showed a strong inverse correlation with clinical laboratory results, specifically blood urea nitrogen and creatinine levels. This pilot study identified plasma exosomal miRNAs, particularly miR-451a and miR-16–2-3p, as potential biomarkers for imatinib resistance in chronic myeloid leukemia. Target gene prediction was performed to explore their regulatory roles. Despite the limited sample size, these findings enhance our understanding of drug resistance mechanisms and warrant further validation in larger cohorts to assess their clinical relevance and therapeutic potential.

## Introduction

Chronic myeloid leukemia (CML) is a myeloproliferative neoplasm originating from multipotent hematopoietic stem cells. The disease is characterized by the presence of the Philadelphia chromosome, which is caused by a reciprocal t(9:22) (q34:q11) translocation that creates the BCR-ABL oncogene encoding a chimeric oncoprotein with constitutive tyrosine kinase activity [1–4]. CML is a hematological malignancy commonly diagnosed in Asia [5]. The development of imatinib mesylate (IM), the first specific BCR-ABL1 inhibitor, has had a major impact on patients with CML [6]. IM treatment induces a complete hematological and cytogenetic response in more than 90 and 80% of newly diagnosed patients with chronic phase CML, respectively, and thus serves as the standard therapy for CML [7]. Although clinical data have shown success with IM treatment, primary resistance to IM and molecular evidence of persistent disease have been detected in 20–30% of IM-treated patients [8,9]. The mechanism of IM resistance has been previously reported, including the presence of point mutations, amplification of the BCR-ABL1 gene [8,10], or abnormal expression of proteins [11,12] or microRNAs (miRNAs) [13–15]. Recently, increasing evidence has suggested that exosome-derived miRNAs are involved in tumor drug resistance and could serve as novel biomarkers for the diagnosis and prediction of drug sensitivity in cancer, especially in hematological malignancies [4,16–18].

Exosomes, the smallest type of extracellular vesicle (EVs), are nanovesicles 40–100 nm in diameter that form within the endosomal compartment and are secreted when a multivesicular body fuses with the plasma membrane [19]. Exosomes can transport a variety of functional molecules, including proteins, lipids, mRNAs, miRNAs, and DNA [20,21]. Additionally, the various substances contained within exosomes are enclosed by a lipid bilayer, which remains stable in body fluids, such as blood, ascites, saliva, and urine [22]. Owing to these unique characteristics, exosomes have become ideal candidate biomarkers for the diagnosis, prognosis, and therapeutic response prediction in many malignant tumors. Previous studies have shown that exosomes are important components of the tumor microenvironment and are implicated in the development of chemoresistance in cancer [4,23,24]. Drug-resistant cancer cells selectively transport miRNAs to EVs for delivery to nearby or distant targets [25]. Regarding IM resistance in CML, previous studies showed that exosomes released by IM-resistant K562 (K562IR) cells and internalized by IM-sensitive cells of the same line (K562IS) exhibited increased survival in the presence of IM. The protein cargo of exosomes derived from K562 and K562IR cells comprised IFITM3, CD146, and CD36, which are specific exosomal markers related to IM resistance [12]. The proteomic profile of exosomes derived from the plasma of IM-resistant and IM-sensitive CML patients has been reported. Specifically, RPL13 and RPL14 were significantly upregulated in IM-resistant CML patients [11]. Furthermore, the role of exosome-derived miRNAs has been reported. Particularly, exosomal miR-365 derived from IM-resistant cells was directly transferred to IM-sensitive cells, conferring IM-resistance traits [26]. Hershkovitz-Rokah et al. reported that miR-30e sensitizes K562 cells and patient primary cells to IM by regulating cell cycle progression between the G1 and S phases [27]. Expression studies have revealed downregulation of miR-199b in patients with CML having a 9q deletion. Lower levels of miR-199b were found in IM-resistant patients, suggesting that it could contribute to drug resistance [28]. Recently, miRNAs involved in exosomal resistance transfer were studied by analyzing miRNA profiles of K562IS and K562IR cell lines, their derived exosomes, and K562IS cells treated with exosomes from resistant cells using microarray analysis. The study found that miR-125b-5p and miR-99a-5p were upregulated, while miR-210-3p and miR-193b-3p were downregulated in K562IR cells, their exosomes, and in K562IS cells treated with K562IR-derived exosomes. Moreover, MDR1 mRNA, a target of miR-193b-3p, showed higher expression in K562IR-derived exosomes and in K562IS cells exposed to these exosomes compared to controls. These findings suggest that MDR1 mRNA may be transferred via exosomes, contributing to imatinib resistance [29,30]. However, single-cell CML cell line models may not fully capture the complexity and heterogeneity of CML *in vivo*. Therefore, it is necessary to analyze exosomal miRNAs from clinical plasma samples to identify reliable biomarkers and potential therapeutic targets for imatinib resistance in CML.

## Materials and methods

### Participants

Peripheral blood samples were collected from CML patients, including IM-sensitive (n = 5) and IM-resistant (n = 5) patients individuals from 23/11/2022 to 29/03/2023. The IM-sensitive and IM-resistant CML groups were defined based on their response to imatinib treatment, following established clinical and molecular criteria. The classification of TKI treatment responses and resistance outcomes in CML patients was conducted in accordance with the European LeukemiaNet (ELN) resistance criteria from 2013 and 2020 [31,32]. Hematological data were assessed using Sysmex XN3000. Biochemical data were also evaluated. This study was performed in accordance with the Declaration of Helsinki and approved by the Ethics Committee of the Faculty of Medicine, Prince of Songkla University (Approval REC:65-015-19-2). Written informed consent was obtained from all participants. Clinical and biological characteristics are summarized in S1 Table.

### Preparation of blood plasma

Peripheral venous blood samples (20 ml) were collected from each participant in the fasting state. Plasma was separated by centrifugation at 2,000 × g for 15 minutes. The plasma supernatant (approximately 10 ml), free of lipidemia and hemolysis, was collected and stored at –80°C for subsequent exosome isolation.

### Isolation of exosomes from plasma by ultracentrifugation (UC)

The samples were diluted 1:1 with cold phosphate-buffered saline (PBS; GIBCO-Invitrogen, Carlsbad, CA, USA) to reduce viscosity and centrifuged at 2,500 × g, 4°C for 15 min to remove cells and cell debris. The supernatant was then centrifuged at 12,000 × g for 45 min to eliminate large particles and apoptotic blebs, followed by filtration through a 0.22-µm pore filter.The supernatant was then transferred to UC tubes and ultracentrifuged at 120,000 × *g*, 4°C for 90 min to enrich exosomes (Beckman Coulter, Optima MAX-XP Ultracentrifuge). The pellet was resuspended in 550 µL of cold PBS for subsequent analysis.

### Purification of exosomes from plasma by size exclusion chromatography (SEC)

SEC was performed according to manufacturer’s instructions. Briefly, 500 µL of exosome sample was loaded into the qEV size exclusion column (qEV2, IZON, Medford, MA) and eluted with PBS. Fractions 1–4 (500 µL each) were pooled and concentrated by ultracentrifugation at 120,000 × g for 90 minutes at 4°C. The exosome pellet was then resuspended in 100 µL of PBS. The particle size and concentration of the exosomes were determined by nanoparticle tracking analysis (NTA).

### Exosome quantification by NTA

The size distribution and concentration of the exosomes were measured using NanoSight NS300 (Malvern Panalytical Ltd., Malvern, UK). Exosomes were diluted with PBS to a concentration of 20 particles per frame. The exosome solutions were illuminated with a 488 nm laser, and the movement of the nanoparticles due to Brownian motion was recorded for 60 s. The camera level was set to 15, and the detection threshold was set to 5. NTA measurements were performed and analyzed from five videos using NanoSight NTA software version 3.2.

### Western blotting

Exosomes were verified by western blotting. Exosome proteins were electrophoresed by 10% sodium dodecyl sulfate-polyacrylamide gel electrophoresis using the TGX Stain-Free™ FastCast™ Acrylamide Kit, 12% (cat#161–0185; Bio-Rad Laboratories, Inc., USA) with the following running conditions: 80 V for 20 min in the stacking gel and 120 V for 1 h in the resolving gel. The separated proteins were transferred onto a PVDF membrane and blocked for 1 h at room temperature with 5% non-fat milk in Tris-buffered saline containing 0.1% Tween-20 (TBS-T). After blocking, the membrane was washed three times with TBS-T for 10 min each and incubated overnight at 4°C with primary antibodies (1:1,000 dilution), including anti-HSP70, anti-CD63, anti-CD9, anti-cytochrome C (Cell Signaling Technology, Inc., MA, USA), and anti-albumin (BioLegend, CA, USA). The membrane was then washed twice with TBS-T for 10 min each, followed by incubation with horseradish peroxidase-conjugated secondary antibodies (1:2,000 dilution) for 2 h at room temperature. After washing, the protein bands were detected using the SuperSignal™ West Dura Extended Duration Substrate (Thermo Fisher Scientific, Waltham, MA, USA) and visualized with a chemiluminescence imager (Alliance Q9 Advanced, UVITEC)

### Transmission electron microscopy (TEM)

The morphology and size of the exosomes were characterized by TEM. Isolated exosome pellets were fixed with 3 μL of 2.5% glutaraldehyde for 30 min at room temperature. Then, 3 μL of each sample was deposited onto carbon/formvar-coated grids and incubated for 10 min. The grids were washed twice with PBS for 3 min, followed by ten washes with distilled water for 2 min each. Each exosome sample was negatively stained with 2.5% uranyl acetate and dried overnight at room temperature. Exosomes were visualized using a transmission electron microscope (JEOL JEM 2010) at 50,000 × magnification at the Scientific Equipment Center of the Prince of Songkla University.

### Small RNA sequencing (RNA-Seq) and data analysis

Total RNA was extracted using the miRNeasy Micro kit (Qiagen, Germany) according to the manufacturer’s protocol and quantitatively assessed using a Nanodrop ND-1000 (Nanodrop; Thermo Fisher Scientific). The library was checked with Qubit® RNA Analysis Kit (Life Technologies), quantified by real-time PCR, and analyzed for size distribution by a bioanalyzer (Agilent Technologies). Quantified libraries were pooled and sequenced on Illumina platforms according to the effective library concentration and amount of data required. RNA-Seq and data collection were performed by Novogene Co., Ltd. Briefly, 3′ and 5′ adaptors were ligated to the 3′ and 5′ ends of small RNA, respectively. First-strand cDNA was then synthesized by hybridization with a reverse transcription primer. A double-stranded cDNA library was generated using PCR enrichment. After purification and size selection, libraries with insertions between 18 and 40 bp were prepared for sequencing using Illumina sequencing with SE50.

### Differential expression of miRNA

Differential expression of miRNAs was determined using DESeq2. P-values were adjusted using the Benjamini–Hochberg method. A corrected P-value of 0.05 was set as the threshold for significantly differential expression by default. For the samples without biological replicates, differential expression analysis of the two samples was performed using edge R. The P-value was adjusted using q-value [33]. Q-value < 0.05 and |log2(foldchange)| > 1 were set as the thresholds for significantly differential expression by default.

### Functional enrichment analysis

Gene Ontology (GO) enrichment analysis was used to identify candidate target genes of the differentially expressed miRNAs. A cluster profile that could adjust for gene length bias was implemented for GO enrichment analysis. Kyoto Encyclopedia of Genes and Genomes (KEGG) [34,35] is a database for understanding the high-level functions and utilities of biological systems, such as cells, organisms, and ecosystems, providing molecular-level information, especially large-scale molecular datasets generated by genome sequencing and other high-throughput experimental technologies (http://www.genome.jp/kegg/). We used the KOBAS [36] software to test the statistical enrichment of target gene candidates in the KEGG pathways.

### Quantitative real-time polymerase chain reaction (PCR)

miR-451a and miR-16–2-3p expression was determined using TaqMan® Small RNA Assays (Thermo Fisher Scientific). miRNA quantitation was performed using a LightCycler® 480 PCR System (Roche Applied Science, Mannheim, Germany). The expression of miRNA was normalized against RNU48 expression and calculated using the 2-^ΔΔ^Ct (comparative Ct) method, with CML sensitive cells as a control. All experiments were performed in triplicate.

### Prediction of miRNA targets

The target genes of the miRNAs were predicted using Target scan [37], miRDB [38], and miRTarBase [39]. To identify the functions of the mRNA targets, enrichment analysis of the miRNA targets was conducted using GO.

### Statistical analysis

To assess the statistical power of our study, a post hoc power analysis was performed using the PS: Power and Sample Size software. Based on the observed effect size, a sample size of 5 per group, an alpha level of 0.05, a delta (mean difference) of 15, and a sigma (standard deviation) of 6, the analysis indicated a statistical power of 0.9. This suggests that our study had a 90% probability of detecting a true effect of this magnitude. Statistical analyses were performed using MedCalc software (free trial version), which provides a comprehensive suite of tools for non-parametric tests, correlation analyses, and data visualization including dot plots and box-and-whisker plots. Comparisons between two groups were conducted using the Mann–Whitney U test, and correlations were assessed by Pearson correlation analysis. Data are presented as means ± standard deviation (SD), and a p-value < 0.05 was considered statistically significant.

## Results

### Quantification and characterization of exosomes

**Fig 1:**
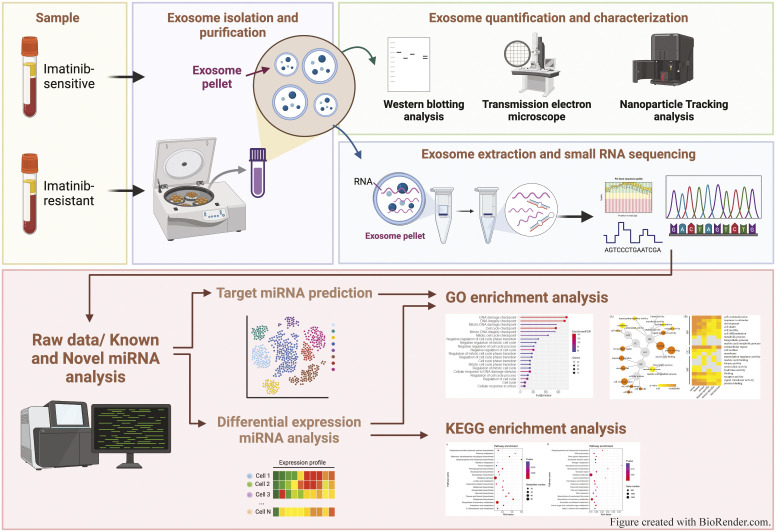
Schematic workflow and bioinformatics pipeline for small RNA analysis of exosome isolated from plasma samples of imatinib-sensitive and imatinib-resistant CML patients. The Figure was created with BioRender.com.

**Fig 2:**
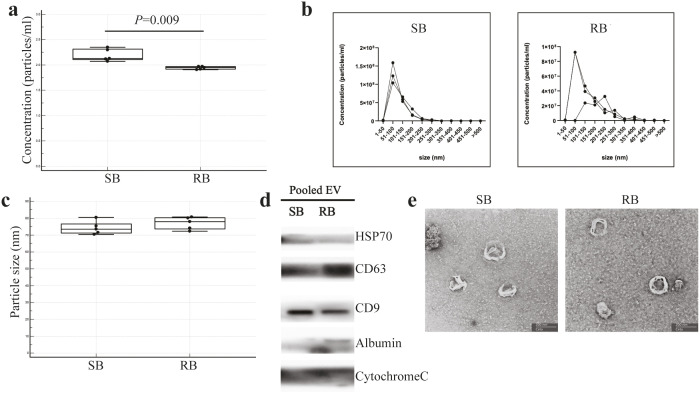
Quantification and characterization of exosomes derived from patients with imatinib sensitive and imatinib resistant CML. Total particle concentration (a), size distribution (b), and particle size (c) in blood plasma derived from imatinib sensitive (sensitive blood; SB) (n = 5) and imatinib resistant (resistant blood; RB) (n = 5) CML patients were determined by Nanoparticle Tracking Analysis (NTA). Characterization of exosomes was performed by western blotting analysis of exosome pellets (d) and TEM analysis of exosomes isolated by ultracentrifugation (e) (magnification: 50,000×). Graphs comparing between two groups are represented as dot pot and box-and-whisker plots. The middle value of the data set is presented as median. The box shows the range of 25^th^–75^th^ percentile. Statistical comparisons between the imatinib-sensitive and imatinib-resistant groups were performed using the non-parametric Mann–Whitney U test.

The study workflow is shown in Fig 1. After isolation by UC and SEC, qualitative and quantitative analyses of total particles in blood plasma derived from the IM-sensitive (sensitive blood; SB) and IM-resistant (resistant blood; RB) groups were performed using NTA. The analysis showed that the concentration of total particles in the IM-sensitive group (n = 5) was 2.19 ± 0.12 × 10^8^ particles/mL, significantly higher than that of the IM-resistant group (n = 5), which was 1.94 ± 0.03 × 10^8^ particles/mL (P = 0.009) (Fig 2a). The size distribution of all particles in the blood ranged from 51 to 100 nm (Fig 2b). Additionally, the mode sizes of the total particles in the plasma of the IM-sensitive and the IM-resistant groups (n = 5) were 74.27 ± 3.93 nm and 77.06 ± 3.73 nm, respectively (Fig 2c). Western blotting analysis revealed that exosome protein markers, including HSP70, CD63, and CD9, were expressed in the isolated exosome samples from both the IM-sensitive and IM-resistant groups. The mitochondrial cytochrome C marker was not detected in the exosome preparations, indicating the absence of other cellular contaminants. Albumin was detected only in the exosomes isolated from the IM-resistant group and appeared as a faint but distinct band (Fig 2d and S1 Fig). Transmission electron microscopy results revealed the presence of typical cup-shaped vesicles with a uniform size of less than 200 nm (Fig 2e).

### Differential expression of exosomal miRNAs between IM-sensitive and IM-resistant CML patients

**Fig 3:**
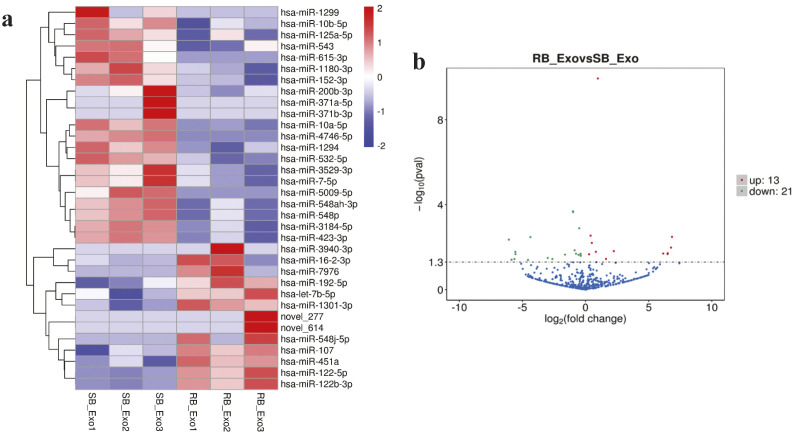
Expression of miRNA in imatinib-sensitive and imatinib-resistant CML patients. (a) Heat map of miRNA expression. (b) Volcano plot of differentially expressed miRNAs. SB, imatinib sensitive blood; RB, imatinib resistant blood; Exo, exosome.

To investigate the differences in the expression of exosomal miRNAs between IM-sensitive and-resistant CML patients, edgeR was used with the following criteria: an adjusted P < 0.05, and a log2 (FC)> or <1. A heatmap generated using hierarchical cluster analysis displayed the miRNA expression levels, which were clearly distinct between IM-sensitive and IM-resistant CML patients (Fig 3a). The statistically significant differentially expressed miRNAs between the two groups are shown as a volcano plot (Fig 3b). We identified 34 miRNAs that were differentially expressed between IM-sensitive and IM-resistant samples, 21 of which were downregulated, while 13 were upregulated (Table 1).

**Table 1:** Exosomal miRNAs differentially upregulated and downregulated in the plasma samples of imatinib-sensitive and imatinib-resistant CML patients.

Expression	miRNA	log2FC	p-value
**Up**	hsa-let-7b-5p	0.401	0.003
	hsa-miR-107	1.618	0.036
	hsa-miR-122-5p	0.281	0.022
	hsa-miR-122b-3p	0.281	0.022
	hsa-miR-1301-3p	2.236	0.015
	***hsa-miR-16–2-3p***	***0.814***	***0.017***
	hsa-miR-192-5p	0.495	0.006
	hsa-miR-3940-3p	6.489	0.021
	***hsa-miR-451a***	***0.972***	***0.000***
	hsa-miR-548j-5p	6.151	0.020
	hsa-miR-7976	6.845	0.003
	novel_277	6.508	0.019
	novel_614	6.765	0.010
**Down**	hsa-miR-10a-5p	−0.499	0.001
	hsa-miR-10b-5p	−0.380	0.025
	hsa-miR-1180-3p	−0.870	0.014
	hsa-miR-125a-5p	−0.555	0.023
	hsa-miR-1294	−2.631	0.036
	hsa-miR-1299	−5.639	0.036
	hsa-miR-152-3p	−4.366	0.003
	hsa-miR-200b-3p	−2.994	0.032
	hsa-miR-3184-5p	−0.382	0.026
	hsa-miR-3529-3p	−0.991	0.000
	hsa-miR-371a-5p	−5.864	0.039
	hsa-miR-371b-3p	−5.864	0.039
	hsa-miR-423-3p	−0.393	0.020
	hsa-miR-4746-5p	−5.566	0.017
	hsa-miR-5009-5p	−6.073	0.004
	hsa-miR-532-5p	−0.760	0.020
	hsa-miR-543	−1.629	0.022
	hsa-miR-548ah-3p	−4.558	0.040
	hsa-miR-548p	−4.558	0.040
	hsa-miR-615-3p	−5.552	0.021
	hsa-miR-7-5p	−0.996	0.000

### Functional enrichment analysis of differentially expressed exosomal miRNAs

**Fig 4:**
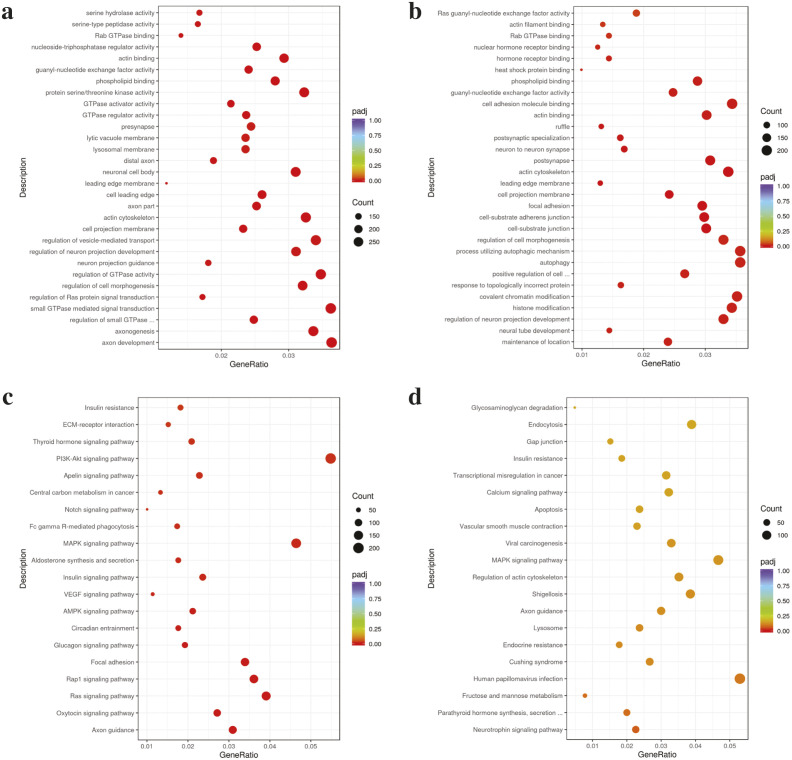
GO and KEGG pathway analyses of differentially expressed miRNAs in imatinib-sensitive and imatinib-resistant CML patients. (a) GO-upregulation and (b) GO-downregulation enrichment analysis. (c) KEGG-upregulation and (d) KEGG-downregulation pathway analysis. GO, Gene ontology; KEGG, Kyoto Encyclopedia of. Genes and Genomes.

We performed GO and KEGG functional enrichment analyses of miRNAs in prognosis-related co-expression RNA networks. GO analysis revealed both upregulation and downregulation of specific terms. For upregulated GO terms, the top three biological process (BP) terms were axon development, small GTPase-mediated signal transduction, and regulation of GTPase activity. The top three cellular component (CC) terms were the actin cytoskeleton, neuronal cell body, and cell leading edge. The top three molecular function (MF) terms were protein serine/threonine kinase activity, actin binding, and phospholipid binding (Fig 4a and S2 Fig). For upregulated GO terms, the top three BP terms were autophagy, processes utilizing the autophagy mechanism, and covalent chromatin modifications. The top three CC terms were actin cytoskeleton, postsynapse, and cell-substrate junction. The top three MF terms were cell adhesion molecule binding, actin binding, and phospholipid binding (Fig 4b and S3 Fig).

KEGG functional enrichment analysis of miRNAs in the prognosis-related co-expression RNA network was also performed. Upregulated KEGG pathways included the PI3K-Akt, MAPK, and RAS signaling pathways (Fig 4c and S4 Fig). Analysis of downregulated KEGG pathways suggested that the differentially expressed miRNAs were involved in the infection of human papillomavirus, MAPK signaling pathway, and shigellosis (Fig 4d and S5 Fig).

### Validation of potential miRNAs by real-time quantitative PCR

**Fig 5:**
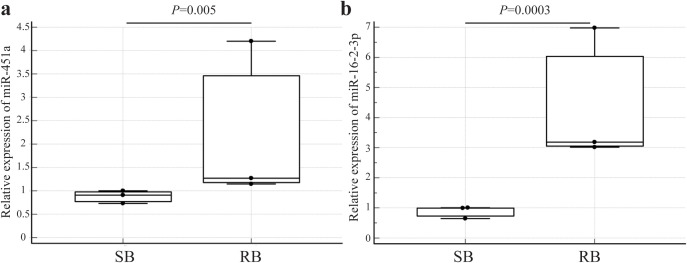
Expression of upregulated exosomal miRNAs. Relative expression of miR-451a (a) and miR-16-2-3p (b) in exosomes derived from the plasma of imatinib-sensitive (SB) (n = 3) and imatinib-resistant (RB) (n = 3) CML patients. Graphs comparing between two groups are represented as dot pot and box-and-whisker plots. The middle value of the data set is presented as median. The box shows the range of 25^th^–75th percentile. Statistical comparisons between the imatinib-sensitive and imatinib-resistant groups were performed using the non-parametric Mann–Whitney U test.

To identify candidate exosomal miRNAs in the IM-sensitive and IM-resistant samples, we randomly selected two candidate miRNAs based on their miRNA expression profiles. The data showed that miR-451a and miR-16–2-3p were upregulated. Specifically, the expression of miR-451a and miR-16–2-3p was higher in the exosomes derived from the blood plasma of IM-resistant samples than in those derived from IM-sensitive samples (Fig 5). This finding is consistent with the RNA-Seq data. Moreover, the correlation between miR-451a and miR-16–2-3p expression levels and exosome numbers was investigated, along with associated clinical and biological characteristics. Our data revealed that miR-451a and miR-16–2-3p expression levels were not correlated with exosome numbers or white blood cell count. Interestingly, miR-16–2-3p expression showed a strong inverse correlation with both blood urea nitrogen (*r* = –0.92, P = 0.010) and creatinine levels (*r* = –0.92, P = 0.009) (S6 Fig).

### Investigation of target genes of miR-451a and miR-16–2-3p

**Fig 6:**
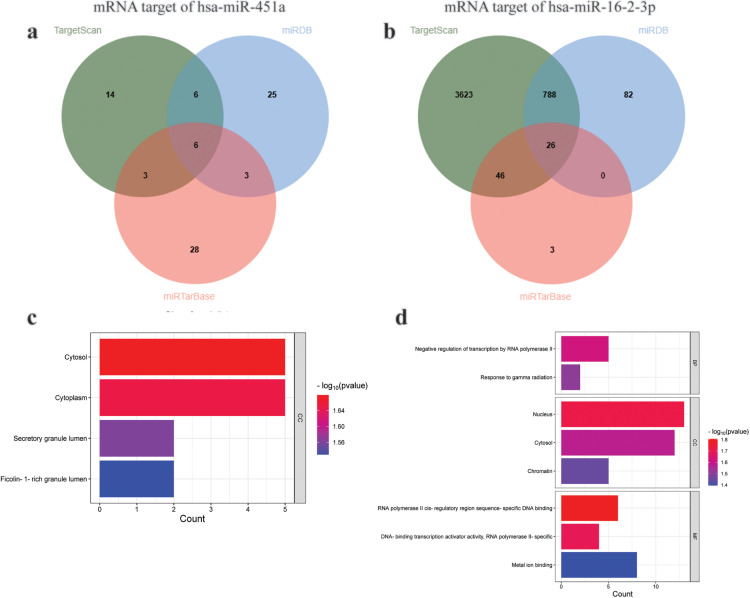
Bioinformatic analysis of mRNA targets of the candidate miRNAs, miR-451a and miR-16-2-3p. The Venn diagram displays the number of predictions of mRNA targets of candidate miRNAs, miR-451a (a) and miR-16-2-3p (b), using TargetScan, miRDB, and miRTarBase. GO enrichment analysis of mRNA targets of the candidate miRNAs, miR-451a (c) and miR-16-2-3p (d).

The mRNA targets of the two candidate miRNAs, miR-451a and miR-16–2-3p, were predicted using TargetScan, miRDB, and miRTarBase. Our results showed that six mRNA targets of miR-451a (ATF2, MIF, CDKN2D, CAB39, OSR1, and PSMB8) were common among the three programs (Fig 6a and S2 Table). We identified the mRNA targets of miR-16–2-3p. A total of 26 potential mRNA targets of miR-16–2-3p were shared across all three programs. Notable candidate mRNA targets of miR-16–2-3p included *RAB1A*, *TM9SF3*, *MYC*, *PRKAA*, *HOXA9*, *USP46*, and *LRIG2* (Fig 6b and S3 Table). GO analysis of the mRNA targets of each miRNA was conducted to predict the functions of the differentially expressed miRNAs. The results are shown in Fig 6c and 6d, respectively.

## Discussion

Several distinct molecular mechanisms underlying IM resistance have been described, and understanding these mechanisms presents a significant clinical challenge in the treatment of CML. Previous reports have identified factors, such as point mutations, BCR-ABL1 overexpression [8,10], abnormal protein expression [11,12], and miRNA dysregulation [13–15] as contributors to IM resistance. Studies have shown that exosomes are linked to drug resistance [4,23,24,40] because exosomes from drug-resistant cancer cells can transfer the resistance features to drug-sensitive cancer cells in various types of tumors [25,41]. In CML, exosomes involved in drug resistance and sensitivity have also been reported [11,12,26–28]. Moreover, exosomes are abundant in biofluids, including blood, urine, saliva, ascitic fluid, and cerebrospinal fluid, making them attractive as potential biomarkers and therapeutic tools. Several studies have identified exosomal protein and miRNA biomarkers with diagnostic significance for drug resistance in hematological malignancies, such as chronic lymphocytic leukemia [42,43], multiple myeloma [44–46], and acute myeloid leukemia [47,48]. The role of plasma exosomes in drug resistance in CML has been reported [11]. Although previous studies have demonstrated differences in exosomal miRNAs between K562IR and K562IS cells, as well as in K562IS cells treated with K562IR-derived exosomes [29], the role of clinical plasma exosomal miRNAs in CML drug resistance has rarely been investigated. In this study, we conducted a comparative analysis of miRNAs in plasma exosomes from imatinib-sensitive and imatinib-resistant CML patients by isolating and characterizing plasma exosomes from these patients.

To the best of our knowledge, this study is the first to report plasma exosome concentrations in IM-sensitive and IM-resistant CML patients. NTA and TEM results showed that the size distribution and morphological structure of plasma exosomes were consistent with those of typical exosomes. Western blot analysis also showed that exosomal markers, such as HSP70, CD63, and CD9, were present in plasma exosomes. These results demonstrate that we effectively isolated exosomes from the plasma of IM-sensitive and IM-resistant CML patients. Subsequently, we performed a differential expression analysis of exosomal miRNAs to identify potential biomarkers associated with IM resistance.

Differential expression analysis of miRNAs between the two groups revealed 34 miRNAs, of which 21 were downregulated and 13 were upregulated. The identified miRNAs differed from those reported in a previous study using miRNA arrays in exosomes derived from K562IS and K562IR cell lines [29]. This discrepancy may be attributed to differences in cell types, as well as the variations between *in vitro* and *in vivo* conditions. GO and KEGG functional analyses showed that the upregulated miRNAs were associated with autophagy, while the downregulated miRNAs were associated with the PI3K-Akt signaling pathway. These differentially expressed exosomal miRNAs illustrate the mechanisms underlying resistance to IM in CML. Previous studies have reported that autophagy and the PI3K-Akt signaling pathway play a role in IM drug resistance in CML [49–52]. To screen miRNA resistance biomarkers, we further analyzed two candidate miRNAs from the miRNA expression profiles. The expression of miR-451a and miR-16–2-3p was upregulated in exosomes derived from the plasma of IM-resistant patients compared with that in IM-sensitive patients. miR-451a, a tumor suppressor, is upregulated in the plasma but downregulated in purified leukocytes from the blood of CML patients [53–56]. In CML-IM resistance, downregulation of miR-451 was observed in peripheral leukocytes [57,58]. However, to the best of our knowledge, this is the first study to identify upregulated expression of miR-451a in exosomes derived from IM-resistant patients. This finding is consistent with the results from plasma samples of CML patients [55]. miR-16, a tumor suppressor, was upregulated in the plasma and purified leukocytes from the blood of CML patients [55,59], but decreased in the chronic phase and blast crisis [60]. In exosomes derived from IM-resistant patients, the expression of miR-16 was increased compared to that in exosomes derived from IM-sensitive patients. Our data suggested that differences in sample sources may affect miRNA expression. Normally, miR-451a and miR-16 are abundant in normal plasma [61], but in CML plasma, both miRNAs are likely upregulated [55]. In plasma exosomes, the expression of miR-451a and miR-16 was elevated, particularly in IM-resistant CML patients. To identify the potential genes and signaling pathways implicated in IM resistance, we analyzed the predicted targets of the candidate miRNAs, miR-451a and miR-16–2-3p, using three different programs: TargetScan, miRDB, and miRTarBase. Among the predicted targets of these miRNAs, we focused on the autophagy and PI3K-Akt signaling pathways, which have been associated with resistance to IM. MIF is a potential target of miR-451. MIF is reportedly a key regulator of tumorigenesis, angiogenesis, and tumor metastasis [62]. MIF also plays a role in drug resistance in acute myeloid leukemia [63] and neuroblastoma cells [64]. MIF has been identified as a target of miR-451 in both nasopharyngeal carcinoma and human colorectal cancer cell lines. This targeting was confirmed using a luciferase reporter assay, demonstrating direct interaction between miR-451 and the 3’ untranslated region of MIF mRNA. Furthermore, western blot analysis revealed that overexpression of miR-451, through the use of a miR-451 mimic, led to a significant decrease in MIF protein levels, indicating post-transcriptional suppression [65,66]. A potential target of miR-16 is RAB1A. RAB proteins, members of the RAS GTPase superfamily, are key regulators of membrane trafficking and fusion events [67]. The role of RAB1 in autophagy involves phagophore assembly via the regulation of ATG9 [68]. In cancer, Rab proteins can play either oncogenic or tumor-suppressive roles, depending on the type of cancer. Previous studies have shown that Rab proteins are involved in various aspects of cancer progression, including migration, invasion, metabolism, exosome secretion, autophagy, and drug resistance [69].

In conclusion, we identified 34 miRNAs in plasma exosomes with significant potential as biomarkers for predicting clinical resistance to IM in CML patients, with exosomal miR-451a and miR-16–2-3p being particularly notable. Moreover, correlation analysis between exosomal miRNAs and clinical parameters revealed that the expression of exosomal miR-16–2-3p and miR-451a was associated with kidney function in IM-resistant patients. These findings suggest that these miRNAs may serve not only as potential biomarkers of drug resistance but also as indicators of renal involvement or systemic toxicity related to IM resistance. While our study identifies associations of miR-451a and miR-16–2-3p with IM resistance, their precise mechanistic roles remain to be elucidated. Future investigations should incorporate functional studies—such as gain- and loss-of-function assays in relevant CML models—to confirm their causal involvement in resistance pathways and to explore their potential as therapeutic targets.

However, this study has several limitations. The data are primarily descriptive, lacking functional validation, and the sample size is limited, largely due to the rarity of this patient population and the challenges in recruiting sufficient eligible participants; however, this is acceptable for a pilot study. Therefore, the current study should be considered exploratory, serving as a pilot investigation to identify candidate exosomal miRNAs associated with IM resistance. Further validation in larger, independent cohorts is warranted to assess their diagnostic and prognostic potential. The absence of healthy controls also limited the ability to determine whether the observed miRNA alterations are specific to CML or reflect broader pathological changes. Future studies, including age- and sex-matched healthy controls, will be essential to validate the specificity and clinical relevance of these miRNAs. Moreover, we did not perform surface marker profiling or apply cell-specific markers to determine the cellular origin of the EVs. Planned follow-up studies will incorporate advanced EV characterization techniques—such as flow cytometry and immunocapture-based surface marker profiling—to address this limitation.

Despite these constraints, our findings provide an initial framework for understanding exosomal miRNA signatures associated with IM resistance in CML and support further investigation into their potential mechanistic roles and clinical applications.

## Supporting information

S1 Fig Unprocessed, full-length western blot membranes used in Fig 2d. (TIF)

S2 Fig GO upregulation analysis of differentially expressed miRNAs in imatinib-sensitive and imatinib-resistant CML patients. (TIF)

S3 Fig GO downregulation analysis of differentially expressed miRNAs in imatinib-sensitive and imatinib-resistant CML patients. (TIF)

S4 Fig KEGG pathway upregulation analysis of differentially expressed miRNAs in imatinib-sensitive and imatinib-resistant CML patients. (TIF)

S5 Fig KEGG pathway downregulation analysis of differentially expressed miRNAs in imatinib-sensitive and imatinib-resistant CML patients. (TIF)

S6 Fig Correlation between miR-451a and miR-16–2-3p expression levels and exosome numbers, as well as clinical and biological characteristics, in imatinib-sensitive and imatinib-resistant CML patients. (TIF)

S1 Table The characteristics of the 10 patients with CML. (DOCX)

S2 Table mRNA targets of hsa-miR-451a were predicted using TargetScan, miRDB, and miRTarBase. (DOCX)

S3 Table mRNA targets of hsa-miR-16–2-3p were predicted using TargetScan, miRDB, and miRTarBase. (DOCX)
