# Supplementary figures and images for: Exosomal miRNA expression profiling in patients with imatinib resistant Chronic myeloid leukemia: A pilot study
Source: PLoS One. 2025 Aug 29;20(8):e0331479. doi: 10.1371/journal.pone.0331479 (PMC12396705; doi:10.1371/journal.pone.0331479)

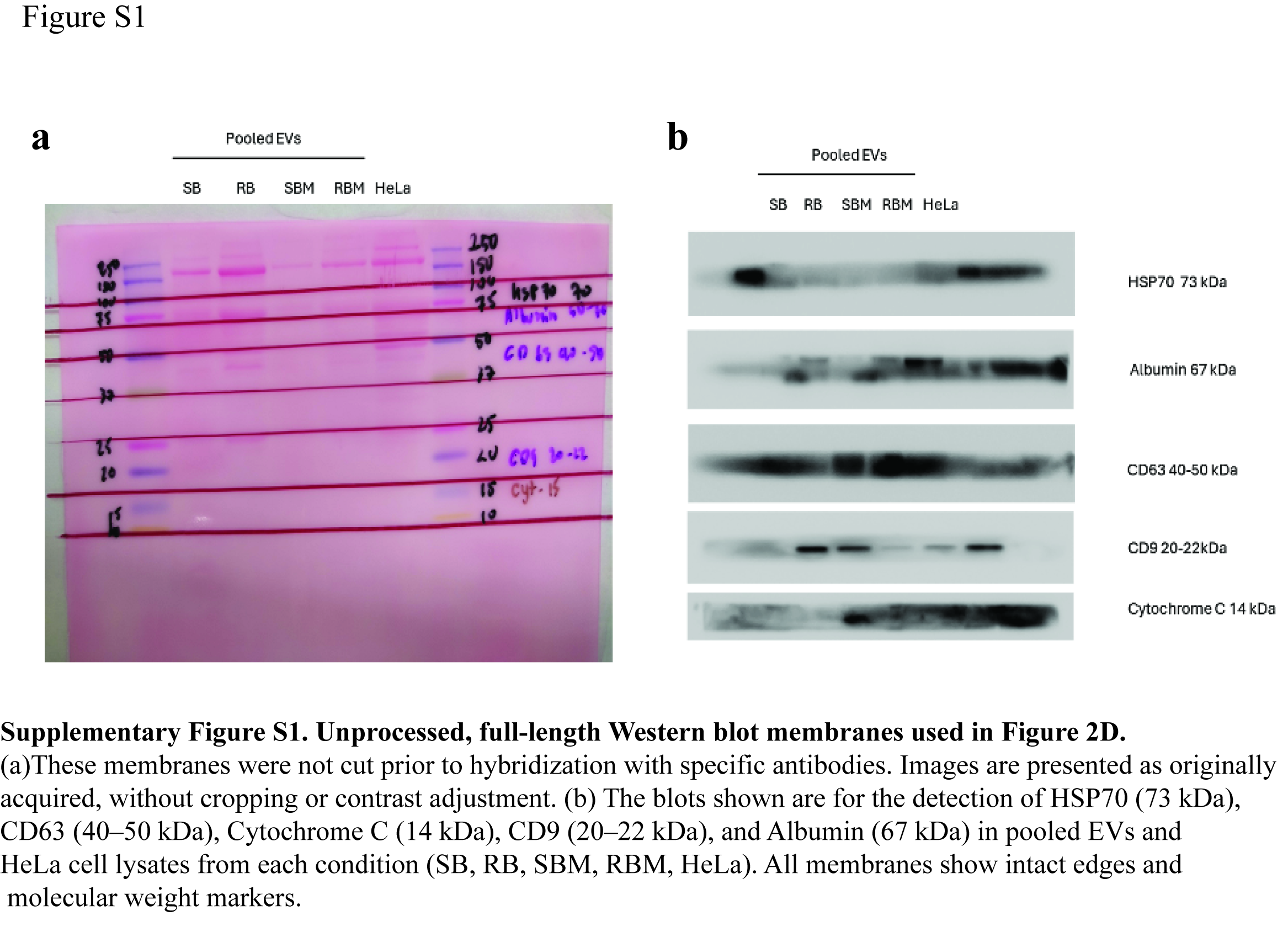

Supplement: S1 Fig — (TIF) [file pone.0331479.s001.tif]

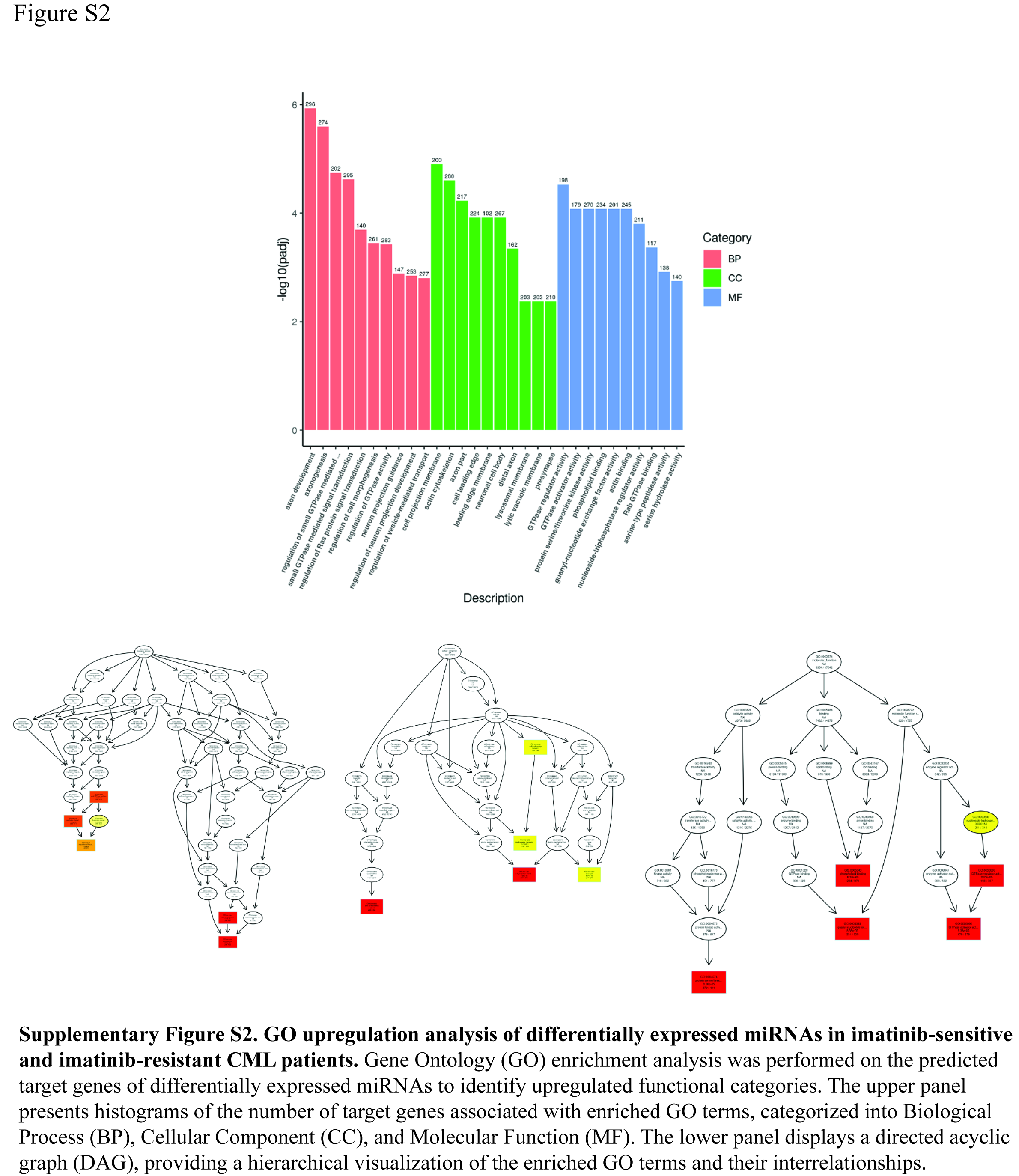

Supplement: S2 Fig — (TIF) [file pone.0331479.s002.tif]

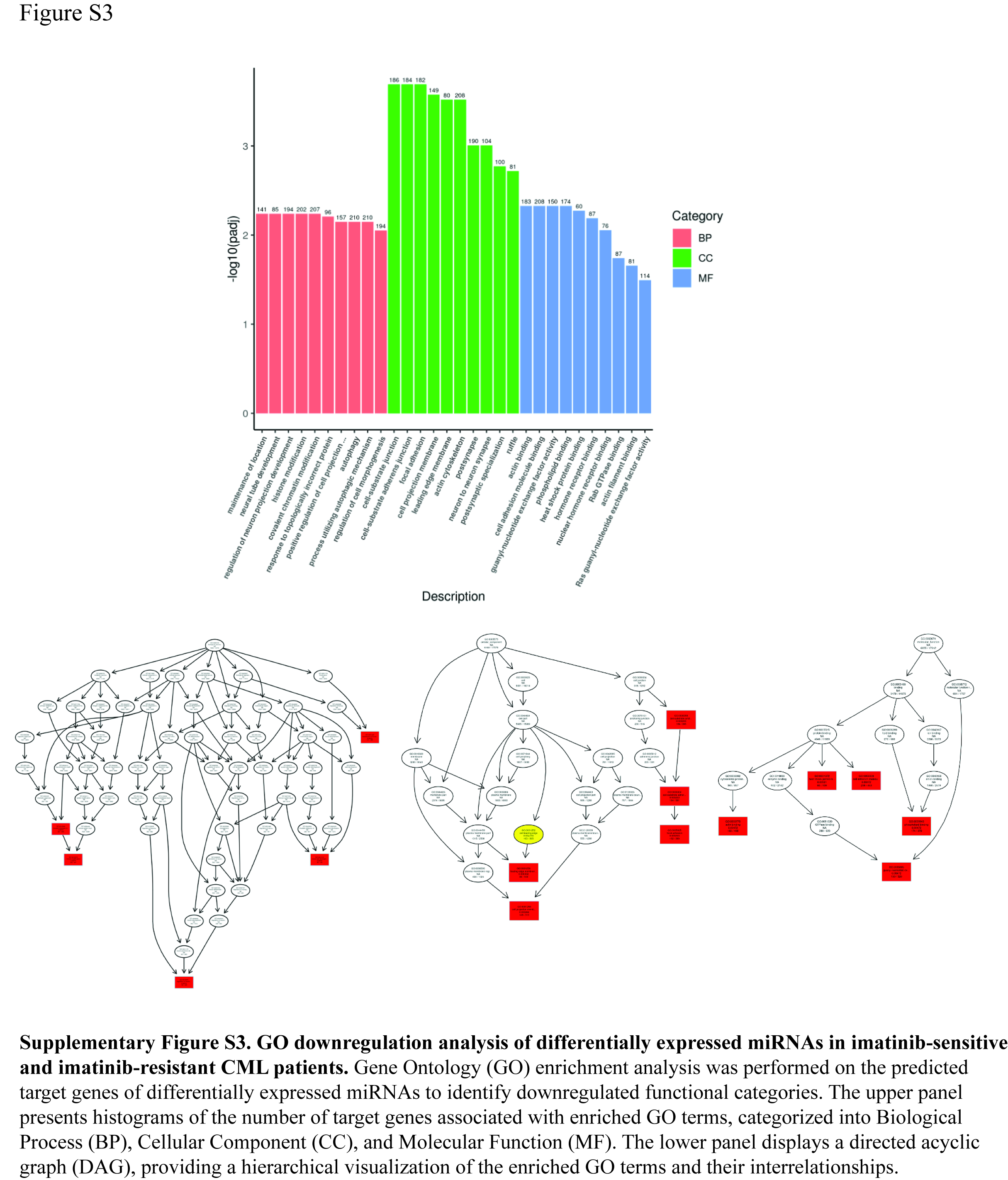

Supplement: S3 Fig — (TIF) [file pone.0331479.s003.tif]

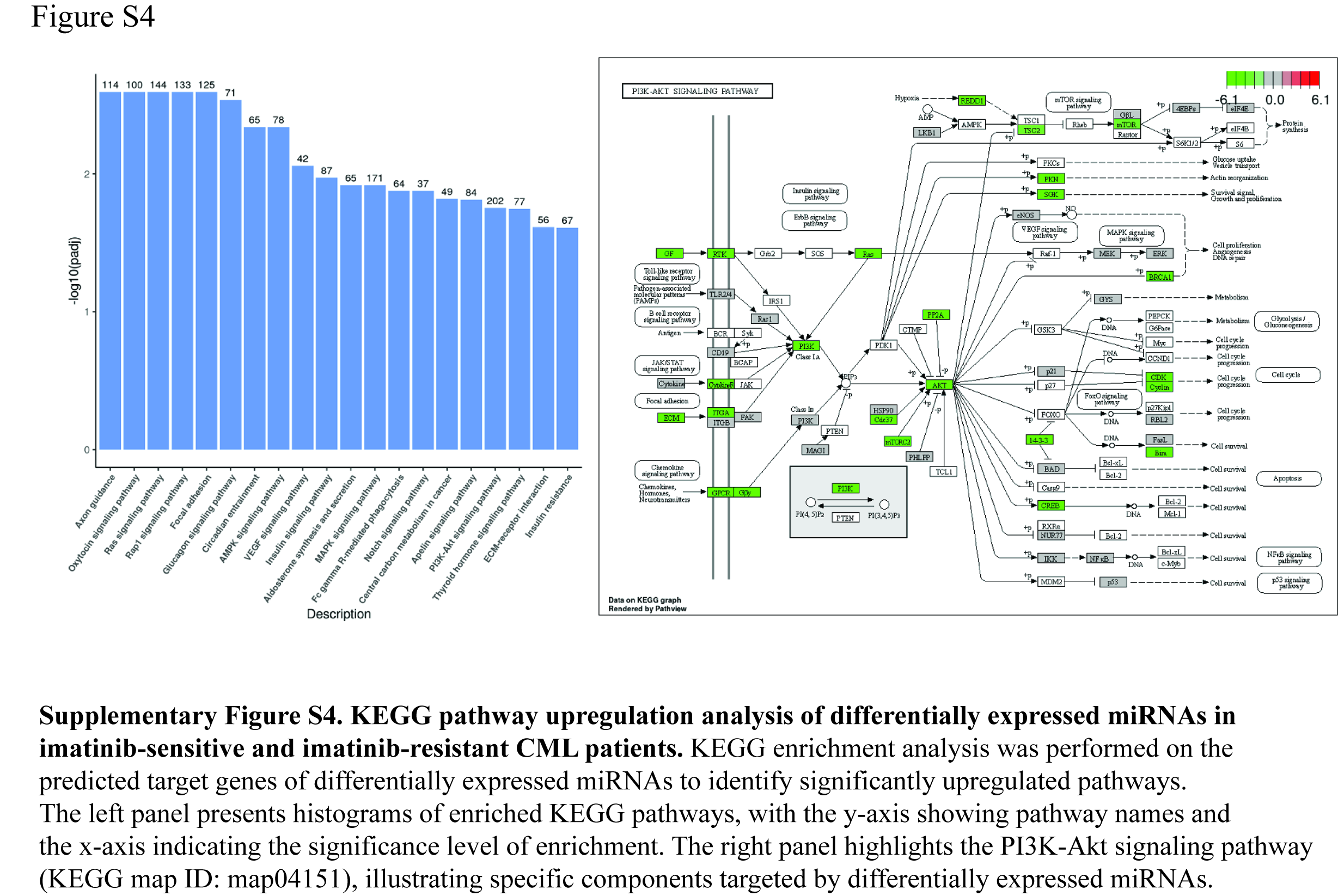

Supplement: S4 Fig — (TIF) [file pone.0331479.s004.tif]

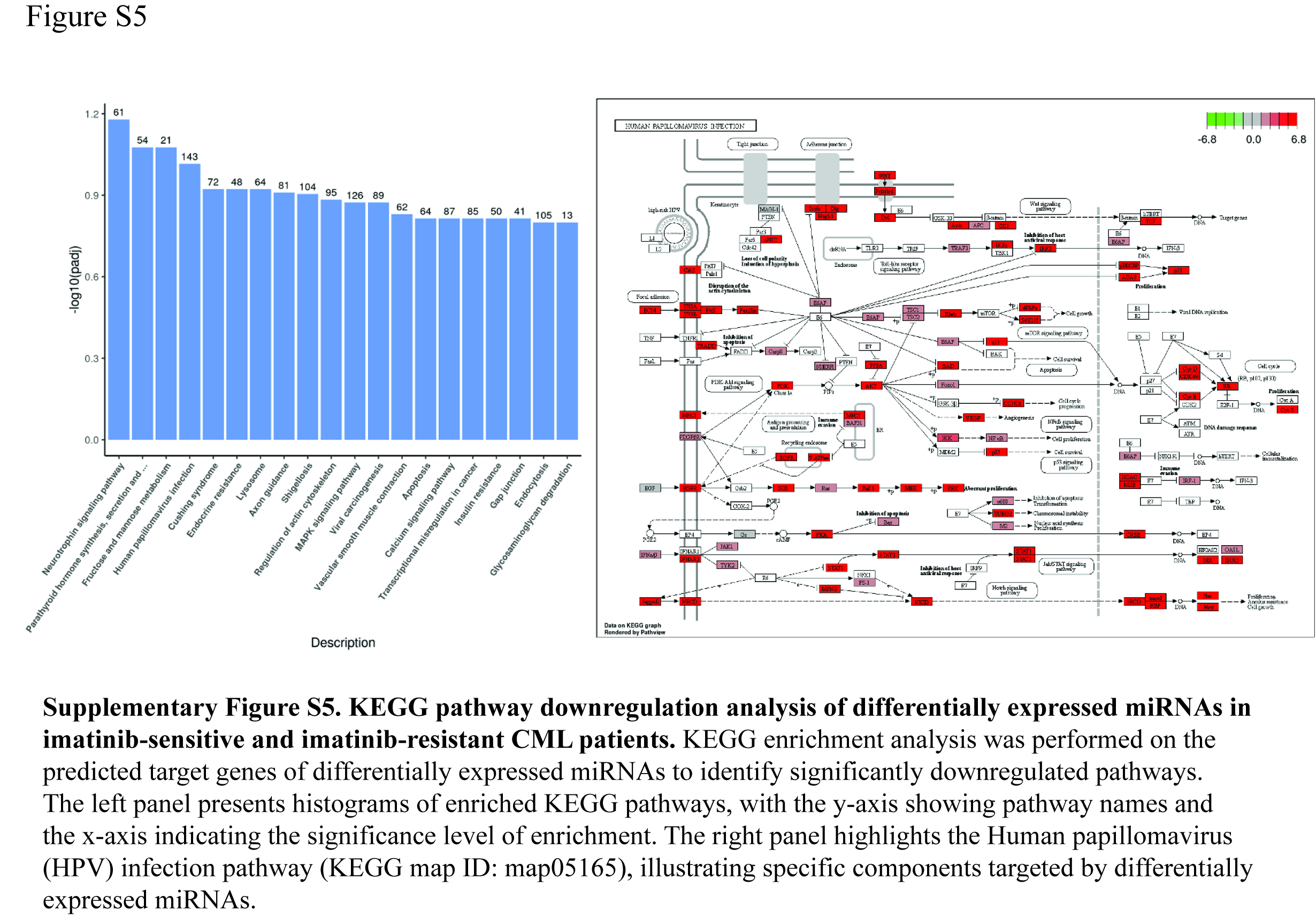

Supplement: S5 Fig — (TIF) [file pone.0331479.s005.tif]

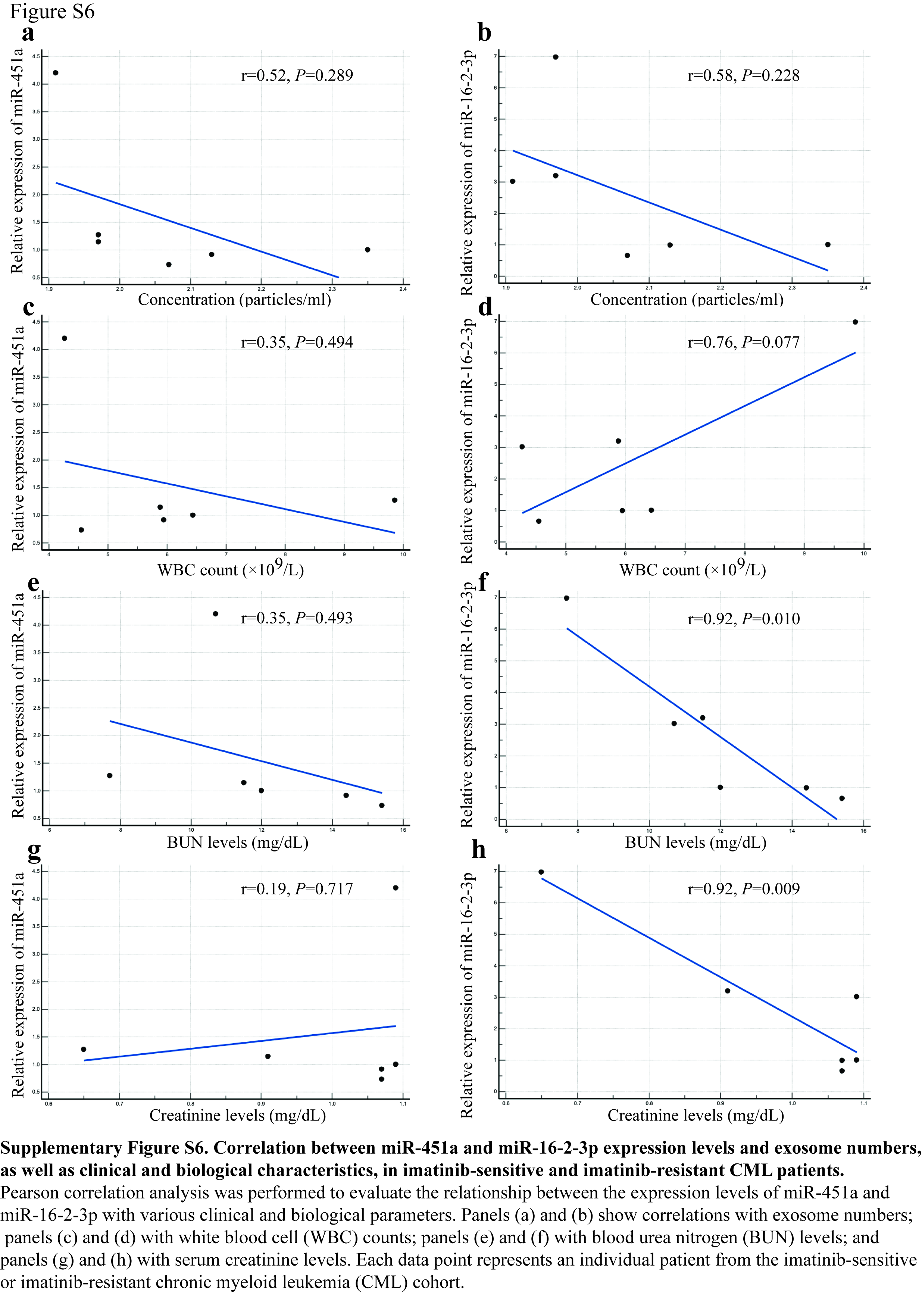

Supplement: S6 Fig — (TIF) [file pone.0331479.s006.tif]
