# Supplementary material for: Exosomal miRNA expression profiling in patients with imatinib resistant Chronic myeloid leukemia: A pilot study
Source: PLoS One. 2025 Aug 29;20(8):e0331479. doi: 10.1371/journal.pone.0331479 (PMC12396705; doi:10.1371/journal.pone.0331479)
Supplement: S1 Table — (DOCX) [file pone.0331479.s007.docx]

**S1 Table. The characteristics of the 10 patients with CML**

| Patient number |  | Gender | Age | WBC count (×10^9^/L) | RBC count (×10^12^/L) | Platelet count (×10^9^/L) | BUN  (mg/dL) | Creatinine (mg/dL) |
| --- | --- | --- | --- | --- | --- | --- | --- | --- |
| 1 | imatinib-sensitive | M | 41 | 4.69 | 3.90 | 270 | 11.3 | 0.78 |
| 2 | imatinib-sensitive | M | 46 | 4.48 | 3.93 | 156 | 17.1 | 1.01 |
| 3 | imatinib-sensitive | M | 49 | 6.44 | 5.2 | 298 | 12 | 0.93 |
| 4 | imatinib-sensitive | M | 74 | 4.55 | 4.16 | 183 | 15.4 | 1.07 |
| 5 | imatinib-sensitive | M | 27 | 5.95 | 5.29 | 269 | 14.4 | 1.07 |
| 6 | imatinib-resistant | M | 80 | 4.27 | 3.76 | 50 | 10.7 | 1.09 |
| 7 | imatinib-resistant | F | 62 | 9.86 | 4.96 | 224 | 7.7 | 0.65 |
| 8 | imatinib-resistant | F | 19 | 6.45 | 3.81 | 215 | 10.2 | 0.58 |
| 9 | imatinib-resistant | M | 36 | 5.89 | 4.62 | 144 | 11.5 | 0.91 |
| 10 | imatinib-resistant | F | 56 | 4.88 | 4.17 | 201 | 9.7 | 0.67 |
