# Supplementary material for: Exosomal miRNA expression profiling in patients with imatinib resistant Chronic myeloid leukemia: A pilot study
Source: PLoS One. 2025 Aug 29;20(8):e0331479. doi: 10.1371/journal.pone.0331479 (PMC12396705; doi:10.1371/journal.pone.0331479)
Supplement: S2 Table — (DOCX) [file pone.0331479.s008.docx]

**S2 Table. mRNA targets of hsa-miR-451a were predicted using TargetScan, miRDB, and miRTarBase**

| **TargetScan** | **miRDB** | **miRTarBase** | **TargetScan\|**  **miRDB** | **TargetScan\|**  **miRTarBase** | **miRDB\|**  **miRTarBase** | **TargetScan\|miRDB\|miRTarBase** |
| --- | --- | --- | --- | --- | --- | --- |
| **C11orf30** | CXCL16 | ABCB1 | S1PR2 | TSC1 | CUX2 | OSR1 |
| **AEBP2** | TARP | AKT1 | VAPA | YWHAZ | CAV1 | ATF2 |
| **GK** | ST8SIA4 | MMP2 | SAMD4B | SZRD1 | RAB5A | MIF |
| **DKFZP779J2370** | FBLN5 | MMP9 | PMM2 |  |  | PSMB8 |
| **TBX1** | CERK | BCL2 | FBXO33 |  |  | CDKN2D |
| **PRICKLE2** | LETM2 | MYC | TTN |  |  | CAB39 |
| **MEF2D** | MEX3C | RAB14 |  |  |  |  |
| **BTBD9** | USP46 | TMED7 |  |  |  |  |
| **TRIM66** | CMTM6 | ARPP19 |  |  |  |  |
| **PRR12** | TBC1D9B | UBE2H |  |  |  |  |
| **GATAD2B** | KIAA1217 | CPNE3 |  |  |  |  |
| **PSMD11** | MAU2 | DCBLD2 |  |  |  |  |
| **VPS18** | RNF217 | IL6R |  |  |  |  |
| **DBNL** | MEGF6 | IKBKB |  |  |  |  |
|  | EVL | FRZB |  |  |  |  |
|  | CDKN2B | PKD1 |  |  |  |  |
|  | UCK1 | ROR2 |  |  |  |  |
|  | C16orf72 | mTOR |  |  |  |  |
|  | DCAF5 | ADAM10 |  |  |  |  |
|  | CACHD1 | CRP |  |  |  |  |
|  | LUZP2 | ETS1 |  |  |  |  |
|  | EIF2AK3 | DGCR8 |  |  |  |  |
|  | AKTIP | ERH |  |  |  |  |
|  | FAM171A1 | HELLS |  |  |  |  |
|  | NEDD9 | CA1 |  |  |  |  |
|  |  | FNTA |  |  |  |  |
|  |  | S1PR3 |  |  |  |  |
|  |  | KDM7A |  |  |  |  |
