# Supplementary material for: Exosomal miRNA expression profiling in patients with imatinib resistant Chronic myeloid leukemia: A pilot study
Source: PLoS One. 2025 Aug 29;20(8):e0331479. doi: 10.1371/journal.pone.0331479 (PMC12396705; doi:10.1371/journal.pone.0331479)
Supplement: S3 Table — (DOCX) [file pone.0331479.s009.docx]

**S3 Table. mRNA targets of hsa-miR-16-2-3p were predicted using TargetScan, miRDB, and miRTarBase**

| **TargetScan\|miRDB** | **TargetScan\|miRTarBase** | **TargetScan\|miRDB\|miRTarBase** |
| --- | --- | --- |
| CADM2 | MTRNR2L6 | B2M |
| BCHE | UBE2E3 | SUB1 |
| RORA | LPP | PABPC4L |
| COMMD8 | RPS4X | RAB1A |
| DGKH | PAPD7 | ZNF431 |
| NAT2 | ANP32E | NUS1 |
| SULT6B1 | HOXA10 | TNPO1 |
| RCN2 | PPIF | PRKAA1 |
| MOB1B | ERGIC2 | ZFX |
| FAM133A | ERBB2IP | HOXA9 |
| GABPB1 | BMI1 | VSNL1 |
| PSMA8 | CCDC14 | RNF219 |
| ATP7A | CTGF | COMMD3-BMI1 |
| ARL15.00 | ALDH1A2 | TM9SF3 |
| TMEM161B | PREPL | PTAR1 |
| NGLY1 | RBBP6 | MYC |
| BCAP29 | CBS | FRK |
| PEX5L | AP3B1 | RPS6KA5 |
| QKI | PTPN14 | KLHL15 |
| USP16 | TMEM117 | AR |
| TSPAN19 | NRIP1 | AMOTL1 |
| EFCAB1 | ACBD5 | USP46 |
| ZNF699 | NFATC2IP | LRIG2 |
| SYT16 | GRPEL2 | RNF44 |
| CCDC179 | TBC1D15 | IPO7 |
| ALDOB | RSBN1 | CLCN3 |
| SPTLC1 | EI24 |  |
| LEAP2 | YWHAG |  |
| CTNNAL1 | CCNT1 |  |
| MYL1 | UBE2D3 |  |
| SPATA9 | NUCKS1 |  |
| CHL1 | IGFBP5 |  |
| TEX30 | ARL6IP1 |  |
| RPE65 | CDC25A |  |
| MSH2 | MRPS30 |  |
| N4BP2 | SULT1B1 |  |
| BDH2 | WASF2 |  |
| CD9 | ATP6V1C1 |  |
| HORMAD1 | ABCG2 |  |
| C2orf66 | DYRK2 |  |
| C14orf39 | AGMAT |  |
| CTTNBP2 | CCT4 |  |
| TUBB2B | SH3GLB1 |  |
| AUH | BID |  |
| GML | SH3BP5 |  |
| PIGP | DROSHA |  |
| CSTF2 |  |  |
| C11orf1 |  |  |
| RGS5 |  |  |
| SNAP47 |  |  |
| TMED7 |  |  |
| C16orf87 |  |  |
| C5AR1 |  |  |
| OR2L13 |  |  |
| HAND2 |  |  |
| CNR1 |  |  |
| MFSD8 |  |  |
| CD200 |  |  |
| KERA |  |  |
| PLAGL1 |  |  |
| LYRM7 |  |  |
| DENND4C |  |  |
| LRRC8B |  |  |
| UBE2I |  |  |
| KLHDC1 |  |  |
| LRRTM4 |  |  |
| DCUN1D5 |  |  |
| FAM181A |  |  |
| FAM114A1 |  |  |
| FGF14 |  |  |
| CCL28 |  |  |
| DNAJB9 |  |  |
| PSMC6 |  |  |
| RPE |  |  |
| CMTM7 |  |  |
| IMMP2L |  |  |
| RFX4 |  |  |
| RAB6C |  |  |
| TCTEX1D2 |  |  |
| ZNF273 |  |  |
| KLF2 |  |  |
| GPRIN3 |  |  |
| SAMD9L |  |  |
| FBXO28 |  |  |
| SLC2A13 |  |  |
| STEAP2 |  |  |
| CCSAP |  |  |
| VDAC1 |  |  |
| CCDC122 |  |  |
| OXCT1 |  |  |
| BTG3 |  |  |
| MEX3B |  |  |
| MARC2 |  |  |
| SUMO3 |  |  |
| HEMGN |  |  |
| AKAP5 |  |  |
| TDG |  |  |
| CREBZF |  |  |
| ZFP36 |  |  |
| C5orf30 |  |  |
| CDK1 |  |  |
| FGF20 |  |  |
| SHQ1 |  |  |
| IMPA1 |  |  |
| CA8 |  |  |
| PYROXD1 |  |  |
| RAB27B |  |  |
| DSTN |  |  |
| STX2 |  |  |
| RPS6KA6 |  |  |
| CXCL6 |  |  |
| ARMCX1 |  |  |
| UBE2B |  |  |
| NAB1 |  |  |
| RSPH4A |  |  |
| ANXA7 |  |  |
| STRN |  |  |
| ZFP30 |  |  |
| RAB6A |  |  |
| ZC3HAV1L |  |  |
| PI4K2B |  |  |
| SLC38A2 |  |  |
| FAM3C |  |  |
| MEI4 |  |  |
| RAB39A |  |  |
| PFDN4 |  |  |
| TRMT10A |  |  |
| OMA1 |  |  |
| CD226 |  |  |
| CIB4 |  |  |
| EXOC6 |  |  |
| PAGE5 |  |  |
| GPATCH2L |  |  |
| C8orf37 |  |  |
| CBLN2 |  |  |
| LPAR1 |  |  |
| PTPN12 |  |  |
| EMB |  |  |
| CMTM8 |  |  |
| HDAC2 |  |  |
| MAP10 |  |  |
| TRMT11 |  |  |
| GALNT1 |  |  |
| SNX25 |  |  |
| KCTD14 |  |  |
| TFEC |  |  |
| AP4E1 |  |  |
| C16orf72 |  |  |
| WNT5A |  |  |
| KRTAP4-5 |  |  |
| PLCXD3 |  |  |
| GINS1 |  |  |
| KLHL4 |  |  |
| ZNF678 |  |  |
| ICK |  |  |
| TNFAIP6 |  |  |
| IGSF11 |  |  |
| PXT1 |  |  |
| CASP3 |  |  |
| RTCA |  |  |
| HSDL1 |  |  |
| ZNF681 |  |  |
| PRRG4 |  |  |
| AEBP2 |  |  |
| PCDHB4 |  |  |
| CCDC82 |  |  |
| GABRG1 |  |  |
| TRIM22 |  |  |
| KIF21A |  |  |
| RNF11 |  |  |
| CUL2 |  |  |
| GOLT1B |  |  |
| SUCLG2 |  |  |
| KIAA1107 |  |  |
| LTV1 |  |  |
| DSC3 |  |  |
| GTF2H3 |  |  |
| LHFPL3 |  |  |
| CNTN4 |  |  |
| CYP26B1 |  |  |
| AIMP1 |  |  |
| USP45 |  |  |
| BET1 |  |  |
| SGIP1 |  |  |
| GPR82 |  |  |
| CCNG2 |  |  |
| TEK |  |  |
| LCA5 |  |  |
| NRG4 |  |  |
| EVA1A |  |  |
| INPP1 |  |  |
| SLC11A2 |  |  |
| C17orf77 |  |  |
| NAPG |  |  |
| GALNT7 |  |  |
| SEPSECS |  |  |
| MLLT11 |  |  |
| PPIL3 |  |  |
| TNFSF4 |  |  |
| COL10A1 |  |  |
| ZNF99 |  |  |
| TMEM33 |  |  |
| PTPN13 |  |  |
| NCAM2 |  |  |
| ID1 |  |  |
| CCT6A |  |  |
| LRRCC1 |  |  |
| ZNF800 |  |  |
| SPOPL |  |  |
| SLC16A7 |  |  |
| ZNF830 |  |  |
| MEP1A |  |  |
| MTF2 |  |  |
| SSX2IP |  |  |
| PIGK |  |  |
| ZNF569 |  |  |
| ZNF280D |  |  |
| RMI1 |  |  |
| SP8 |  |  |
| PCDHB13 |  |  |
| DNAJB14 |  |  |
| HFM1 |  |  |
| NR5A2 |  |  |
| ZNF260 |  |  |
| MIER3 |  |  |
| KIAA1109 |  |  |
| ACER3 |  |  |
| INTU |  |  |
| BAALC |  |  |
| IRF6 |  |  |
| VEGFC |  |  |
| PCGF3 |  |  |
| FAM126B |  |  |
| ZDHHC17 |  |  |
| PPP1R3A |  |  |
| DPY19L4 |  |  |
| TP53BP2 |  |  |
| CYP1B1 |  |  |
| RGMB |  |  |
| PURA |  |  |
| FAM91A1 |  |  |
| RBM25 |  |  |
| CPS1 |  |  |
| PLN |  |  |
| ZBTB21 |  |  |
| SS18 |  |  |
| HNRNPLL |  |  |
| HNF4G |  |  |
| C5orf24 |  |  |
| OAZ1 |  |  |
| PRMT3 |  |  |
| GDPD1 |  |  |
| PLAUR |  |  |
| PAN3 |  |  |
| EBF3 |  |  |
| ZBTB44 |  |  |
| MBIP |  |  |
| ATP6V0A4 |  |  |
| CAV2 |  |  |
| RAP1A |  |  |
| FRS2 |  |  |
| CCDC117 |  |  |
| POLR1B |  |  |
| PREX2 |  |  |
| KIF18A |  |  |
| BBS10 |  |  |
| HIRA |  |  |
| SOAT1 |  |  |
| STAG2 |  |  |
| TPK1 |  |  |
| NEK7 |  |  |
| HMGCS2 |  |  |
| SEMA3E |  |  |
| NACC1 |  |  |
| PTPN4 |  |  |
| ZNF713 |  |  |
| SRSF10 |  |  |
| ITGAV |  |  |
| DCAF8L1 |  |  |
| PRKG1 |  |  |
| PLAG1 |  |  |
| BCAS2 |  |  |
| CLTC |  |  |
| THAP9 |  |  |
| KIAA0408 |  |  |
| YWHAZ |  |  |
| VAMP7 |  |  |
| C2orf73 |  |  |
| DKK1.00 |  |  |
| PRG4 |  |  |
| C1orf21 |  |  |
| GPR37 |  |  |
| HPN |  |  |
| CDH9 |  |  |
| ITGB8 |  |  |
| CPEB2 |  |  |
| TMEM30A |  |  |
| EPM2AIP1 |  |  |
| KBTBD3 |  |  |
| SOGA3 |  |  |
| CHIC1 |  |  |
| SLC12A1 |  |  |
| CAMK1D |  |  |
| FNDC1 |  |  |
| ITPRIPL2 |  |  |
| UFL1 |  |  |
| TMEM168 |  |  |
| RAB11FIP2 |  |  |
| TMEM50A |  |  |
| RGS4 |  |  |
| PBLD |  |  |
| BCOR |  |  |
| SPRED1 |  |  |
| OSM |  |  |
| TMX1 |  |  |
| CCDC36 |  |  |
| ST6GAL1 |  |  |
| LGSN |  |  |
| KIT |  |  |
| FGFR1OP |  |  |
| PTER |  |  |
| ZNF615 |  |  |
| GABPA |  |  |
| HIPK1 |  |  |
| MPP5 |  |  |
| MTMR6 |  |  |
| CCDC30 |  |  |
| FAM111A |  |  |
| GMFB |  |  |
| MSI2 |  |  |
| ZNF195 |  |  |
| LRRC40 |  |  |
| ZNF181 |  |  |
| CCDC85A |  |  |
| C10orf126 |  |  |
| SOST |  |  |
| CDK17 |  |  |
| ATP6V1C2 |  |  |
| MIPOL1 |  |  |
| ZW10 |  |  |
| DGKE |  |  |
| CDH8 |  |  |
| SLC4A7 |  |  |
| CLOCK |  |  |
| HLTF |  |  |
| TMTC1 |  |  |
| ARAP2 |  |  |
| ACKR3 |  |  |
| SV2A |  |  |
| C3orf80 |  |  |
| FSD1L |  |  |
| ZNF780B |  |  |
| PLSCR4 |  |  |
| LRIG1 |  |  |
| NYAP2 |  |  |
| COL23A1 |  |  |
| CNTN1 |  |  |
| SPCS3 |  |  |
| SFPQ |  |  |
| PHLPP1 |  |  |
| LAMP2 |  |  |
| RAB39B |  |  |
| SNRNP48 |  |  |
| RAI14 |  |  |
| PCK1 |  |  |
| KL |  |  |
| CNTNAP3B |  |  |
| HSPA5 |  |  |
| GABRA5 |  |  |
| SMIM14 |  |  |
| MDM4 |  |  |
| PAIP2 |  |  |
| ABCE1 |  |  |
| RAB23 |  |  |
| DARS |  |  |
| ZNF770 |  |  |
| DCLK1 |  |  |
| GFPT1 |  |  |
| PAFAH1B1 |  |  |
| BARD1 |  |  |
| AGL |  |  |
| KCNMB2 |  |  |
| GPALPP1 |  |  |
| AKAP11 |  |  |
| ZNF705A |  |  |
| ACTN1 |  |  |
| TAF15 |  |  |
| N4BP1 |  |  |
| PDZD8 |  |  |
| CDK6 |  |  |
| PIK3R1 |  |  |
| GRIP1 |  |  |
| THSD7B |  |  |
| PDLIM5 |  |  |
| ZNF420 |  |  |
| FBXO45 |  |  |
| BROX |  |  |
| PRRX1 |  |  |
| ZNF423 |  |  |
| IQCH |  |  |
| SPATA6 |  |  |
| TMEM155 |  |  |
| MTF1 |  |  |
| FPGT |  |  |
| ZNF10 |  |  |
| IYD |  |  |
| XK |  |  |
| TMLHE |  |  |
| BLOC1S2 |  |  |
| TRPS1 |  |  |
| RND3 |  |  |
| ZNF746 |  |  |
| HIF1A |  |  |
| DLAT |  |  |
| PRLR |  |  |
| MTHFD2L |  |  |
| AKAP7 |  |  |
| PDE10A |  |  |
| MYEF2 |  |  |
| PICALM |  |  |
| C12orf66 |  |  |
| ARPP19 |  |  |
| FAM49B |  |  |
| NSL1 |  |  |
| ATP2C1 |  |  |
| BRPF1 |  |  |
| NAA30 |  |  |
| ETV1 |  |  |
| TSHZ2 |  |  |
| FOXR2 |  |  |
| TP63 |  |  |
| NEGR1 |  |  |
| FAM126A |  |  |
| ZNF117 |  |  |
| AHCTF1 |  |  |
| CCDC89 |  |  |
| CRISPLD1 |  |  |
| CCP110 |  |  |
| CCDC141 |  |  |
| ZNF254 |  |  |
| GNAS |  |  |
| TERF1 |  |  |
| ERAP1 |  |  |
| PHF6 |  |  |
| B3GALT2 |  |  |
| ATAD1 |  |  |
| CLPX |  |  |
| CDH12 |  |  |
| NBEAL1 |  |  |
| THAP2 |  |  |
| SLC26A4 |  |  |
| INO80D |  |  |
| GNG2 |  |  |
| SEC24A |  |  |
| MAP3K5 |  |  |
| BIRC3 |  |  |
| MCCC2 |  |  |
| CREB1 |  |  |
| WDR89 |  |  |
| TSHZ1 |  |  |
| KLHL5 |  |  |
| FMNL2 |  |  |
| AMIGO2 |  |  |
| CYBB |  |  |
| BTG1 |  |  |
| PGAP1 |  |  |
| TMEM38B |  |  |
| PGR |  |  |
| KDM1B |  |  |
| CMPK1 |  |  |
| NAPEPLD |  |  |
| RNF6 |  |  |
| NFYB |  |  |
| LYPLA1 |  |  |
| TRAF5 |  |  |
| SMAP2 |  |  |
| DNM3 |  |  |
| HS2ST1 |  |  |
| RAB22A |  |  |
| SASS6 |  |  |
| DGKI |  |  |
| RFT1 |  |  |
| STRBP |  |  |
| CCDC50 |  |  |
| BCL2L11 |  |  |
| ACP1 |  |  |
| TTC33 |  |  |
| NCAPG2 |  |  |
| ZNF140 |  |  |
| RNF111 |  |  |
| RB1CC1 |  |  |
| ETF1 |  |  |
| IDI1 |  |  |
| PPM1L |  |  |
| CLIC5 |  |  |
| EIF1AX |  |  |
| PCDH11Y |  |  |
| CHN1 |  |  |
| RC3H1 |  |  |
| TRIM33 |  |  |
| KCTD15 |  |  |
| CNTNAP3 |  |  |
| MBTD1 |  |  |
| ZNF674 |  |  |
| GRAP2 |  |  |
| TRPM7 |  |  |
| MED14 |  |  |
| JUNB |  |  |
| HNRNPAB |  |  |
| ANKRD13C |  |  |
| PHIP |  |  |
| CMKLR1 |  |  |
| TAT |  |  |
| SEMA5A |  |  |
| APBB2 |  |  |
| TEX2 |  |  |
| CDC42BPB |  |  |
| ANKRD44 |  |  |
| DLL1 |  |  |
| CNTNAP2 |  |  |
| ANKRD26 |  |  |
| IFIT2 |  |  |
| TES |  |  |
| PCLO |  |  |
| OLFML2B |  |  |
| MAGT1 |  |  |
| TANC2 |  |  |
| ITGA2 |  |  |
| DSTYK |  |  |
| PEAK1 |  |  |
| GRIA2 |  |  |
| STON2 |  |  |
| THRB |  |  |
| OPHN1 |  |  |
| PRDM15 |  |  |
| DST |  |  |
| GSE1 |  |  |
| OLA1 |  |  |
| PPP2R5C |  |  |
| SLC2A12 |  |  |
| EPN3 |  |  |
| PKD2 |  |  |
| MBOAT2 |  |  |
| INPP4A |  |  |
| LARP4 |  |  |
| ACOX1 |  |  |
| PBRM1 |  |  |
| SOCS5 |  |  |
| NAIP |  |  |
| PHF20 |  |  |
| CNOT7 |  |  |
| CACNA2D1 |  |  |
| SNX18 |  |  |
| IL13RA1 |  |  |
| PHC3 |  |  |
| NOVA1 |  |  |
| GDI2 |  |  |
| AAK1 |  |  |
| SCAI |  |  |
| ETNK1 |  |  |
| ZNF451 |  |  |
| PSTK |  |  |
| ARHGAP12 |  |  |
| DNAJC10 |  |  |
| EIF4E3 |  |  |
| OGFRL1 |  |  |
| RUNX2 |  |  |
| DENND1B |  |  |
| LLGL1 |  |  |
| PPP1R12A |  |  |
| CDC73 |  |  |
| MTSS1 |  |  |
| PRDM8 |  |  |
| DLG2 |  |  |
| PRDM10 |  |  |
| ARID5B |  |  |
| PTPRN2 |  |  |
| KAT6B |  |  |
| NOL10 |  |  |
| SDK2 |  |  |
| RAD21 |  |  |
| ZNF236 |  |  |
| PPP1R12B |  |  |
| SYVN1 |  |  |
| RNF144A |  |  |
| CLASP2 |  |  |
| MAP3K2 |  |  |
| TPP2 |  |  |
| LPGAT1 |  |  |
| ACSL6 |  |  |
| DNAJC3 |  |  |
| SH3D19 |  |  |
| RTN4IP1 |  |  |
| CPNE8 |  |  |
| FAM20B |  |  |
| ZNF41 |  |  |
| OR2C3 |  |  |
| TBX18 |  |  |
| PDE5A |  |  |
| GRSF1 |  |  |
| YTHDF3 |  |  |
| MME |  |  |
| SP3 |  |  |
| EXPH5 |  |  |
| HP1BP3 |  |  |
| SLC26A2 |  |  |
| CYYR1 |  |  |
| CALD1 |  |  |
| C18orf54 |  |  |
| TLK1 |  |  |
| INSR |  |  |
| TENM1 |  |  |
| HS6ST2 |  |  |
| SNRK |  |  |
| IL16 |  |  |
| TRPM3 |  |  |
| UBP1 |  |  |
| EFR3A |  |  |
| ZNF536 |  |  |
| ZNF558 |  |  |
| HECTD4 |  |  |
| CPNE3 |  |  |
| GOLIM4 |  |  |
| AFF2 |  |  |
| ELAVL2 |  |  |
| RAB6B |  |  |
| ARHGEF6 |  |  |
| CDON |  |  |
| MSN |  |  |
| THOC2 |  |  |
| ZZZ3 |  |  |
| MBD5 |  |  |
| HAUS2 |  |  |
| VPS37B |  |  |
| MAPK9 |  |  |
| QSOX2 |  |  |
| CHML |  |  |
| PDP2 |  |  |
| KAT6A |  |  |
| ZNF107 |  |  |
| RAB3C |  |  |
| LRPPRC |  |  |
| CPEB3 |  |  |
| CNTN3 |  |  |
| USP8 |  |  |
| LRRC8C |  |  |
| ZBTB38 |  |  |
| NFATC2 |  |  |
| ATP2B3 |  |  |
| CACUL1 |  |  |
| MAP4K3 |  |  |
| SAMD5 |  |  |
| PKHD1 |  |  |
| RP2 |  |  |
| TTC9 |  |  |
| EPB41L5 |  |  |
| ZSWIM6 |  |  |
| ELK4 |  |  |
| SHANK2 |  |  |
| POGK |  |  |
| DIP2B |  |  |
| TBC1D12 |  |  |
| PRDM5 |  |  |
| INTS6 |  |  |
| ZNF106 |  |  |
| TCERG1L |  |  |
| SECISBP2L |  |  |
| ARHGEF12 |  |  |
| PRDM6 |  |  |
| TMBIM4 |  |  |
| PIAS2 |  |  |
| TFCP2L1 |  |  |
| ZFP82 |  |  |
| ACVR2A |  |  |
| SNX2 |  |  |
| GNB4 |  |  |
| ZNF471 |  |  |
| CDK13 |  |  |
| TAB3 |  |  |
| CFL2 |  |  |
| BTRC |  |  |
| PPP1R3C |  |  |
| FBXO32 |  |  |
| ATRX |  |  |
| WDHD1 |  |  |
| RBM27 |  |  |
| GTF3C2 |  |  |
| PCDHA2 |  |  |
| VEZF1 |  |  |
| CTNND2 |  |  |
| MED13 |  |  |
| PCDHA9 |  |  |
| ABTB2 |  |  |
| RBFOX1 |  |  |
| ADCYAP1 |  |  |
| HTR2C |  |  |
| RBL1 |  |  |
| SLC39A14 |  |  |
| ATP2B1 |  |  |
| ATG12 |  |  |
| FAM122B |  |  |
| MON2 |  |  |
| ARHGAP23 |  |  |
| SREK1 |  |  |
| PCDH7 |  |  |
| OSBPL8 |  |  |
| RUNX1 |  |  |
| DOCK5 |  |  |
| ZNF507 |  |  |
| AGPAT4 |  |  |
| WDR72 |  |  |
| MBNL2 |  |  |
| REPS2 |  |  |
| MED13L |  |  |
| FMNL3 |  |  |
| COL11A1 |  |  |
| GPATCH2 |  |  |
| ARFGEF2 |  |  |
| CCDC173 |  |  |
| ARL1.00 |  |  |
| SCN2A |  |  |
| ZNF662 |  |  |
| YME1L1 |  |  |
| DDX31 |  |  |
| PMPCB |  |  |
| FGL2 |  |  |
| SOD2 |  |  |
| ARFGEF1 |  |  |
| FNDC3B |  |  |
| SNIP1 |  |  |
| FGF9 |  |  |
| RANBP3 |  |  |
| WWP2 |  |  |
| TPR |  |  |
| EPHB4 |  |  |
| SNX27 |  |  |
| MOXD1 |  |  |
| SEMA3C |  |  |
| COX15 |  |  |
| DBF4 |  |  |
| TMEM248 |  |  |
| SENP6 |  |  |
| ZNF566 |  |  |
| TOX3 |  |  |
| AZIN1 |  |  |
| ACSL3 |  |  |
| SCARB2 |  |  |
| SSPN |  |  |
| TNKS2 |  |  |
| MRPL42 |  |  |
| SDE2 |  |  |
| TRIM13 |  |  |
| DNAJC21 |  |  |
| RRAS2 |  |  |
| UBR5 |  |  |
| STX3 |  |  |
| AGPS |  |  |
| HNRNPA0 |  |  |
| ZFAND1 |  |  |
| TUBB |  |  |
| CD59 |  |  |
| ZMYM4 |  |  |
| TSPYL1 |  |  |
| ARHGAP29 |  |  |
| C1orf61 |  |  |
| CLEC12B |  |  |
| SIN3B |  |  |
| CEP63 |  |  |
| TBC1D3H |  |  |
| GIMAP6 |  |  |
| C14orf119 |  |  |
| BCL11A |  |  |
| DCC |  |  |
| CDH19 |  |  |
| COG6 |  |  |
| TRDN |  |  |
| CD36 |  |  |
| SLAMF8 |  |  |
| RBM46 |  |  |
| PLG |  |  |
| GABRR2 |  |  |
| TCP1 |  |  |
| PCDH11X |  |  |
| CERS5 |  |  |
| RSPO3 |  |  |
| USP48 |  |  |
| RGS2 |  |  |
| ESRP2 |  |  |
